# Encouraging residents’ professional development and career planning: the role of a development-oriented performance assessment

**DOI:** 10.1186/s12909-018-1317-9

**Published:** 2018-09-05

**Authors:** Kirsten Dijkhuizen, Jacqueline Bustraan, Arnout J. de Beaufort, Sophie I. Velthuis, Erik W. Driessen, Jan M. M. van Lith

**Affiliations:** 1 Centre for Innovation in Medical Education, Leiden University Medical Centre, PO Box 9600, Zone V7-P, 2300 RC Leiden, the Netherlands; 2 Department of Obstetrics, Leiden University Medical Centre, PO Box 9600, Zone K6-P, 2300 RC Leiden, the Netherlands; 3 Department of Educational Development & Research Maastricht University, Universiteitssingel 60, 6229 Maastricht, the Netherlands

**Keywords:** Postgraduate medical education, Generic competencies, Reflective practice, Deliberate practice, Professional development, Performance assessment, Development Centre, Assessment Centre, Career planning, Qualitative research

## Abstract

**Background:**

Current postgraduate medical training programmes fall short regarding residents’ development of generic competencies (communication, collaboration, leadership, professionalism) and reflective and deliberate practice. Paying attention to these non-technical skills in a structural manner during postgraduate training could result in a workforce better prepared for practice. A development-oriented performance assessment (PA), which assists residents with assessment of performance and deliberately planned learning activities, could potentially contribute to filling this gap. This study aims to explore residents experiences with the PA.

**Methods:**

We conducted a qualitative interview study with 16 residents from four different medical specialties who participated in the PA, scheduled halfway postgraduate training. The PA was conducted by an external facilitator, a psychologist, and focused specifically on professional development and career planning. Residents were interviewed 6 months after the PA. Data were analysed using the framework method for qualitative analysis.

**Results:**

Residents found the PA to be of additional value for their training. The overarching merit was the opportunity to evaluate competencies not usually addressed in workplace-based assessments and progress conversations. In addition, the PA proved a valuable tool for assisting residents with reflecting upon their work and formulating their learning objectives and activities. Residents reported increased awareness of capacity, self-confidence and enhanced feelings of career-ownership. An important factor contributing to these outcomes was the relationship of trust with the facilitator and programme director.

**Conclusion:**

The PA is a promising tool in fostering the development of generic competencies and reflective and deliberate practice. The participating residents, facilitator and programme directors were able to contribute to a safe learning environment away from the busy workplace. The facilitator plays an important role by providing credible and informative feedback. Commitment of the programme director is important for the implementation of developmental plans and learning activities.

## Background

With the increasingly complex and dynamic health care systems, health care professionals face the challenging task of functioning optimally in such environments. In order to thrive, these professionals need to engage in lifelong learning and they need to possess the capacity to quickly adapt to changing circumstances [1]. Both largely depend on: 1) the mastery of generic competencies (communication, collaboration, leadership, professionalism) and 2) the ability to reflect. However, the development of these competencies is often underrepresented in current postgraduate medical training programs.

An effective strategy for lifelong learning and continuous improvement of competencies is deliberate practice [2, 3]. Deliberate practice is defined as “highly structured activities explicitly directed at improvement of performance in a particular domain” and is associated with expert performance. It entails several important aspects: a well-defined task, repeated engagement in executing said task, sufficient resources, detailed and immediate feedback on performance and a motivated and reflective learner [2, 3]. However, in practice, workplace learning by residents and specialists is often reactive, implicit and mainly driven by patient care instead of directed by developmental learning goals and deliberately planned activities [4].

Another feature of workplace learning is that both residents and their supervisors primarily focus on medical knowledge and technical skills. In contrast, learning goals related to generic competencies, such as communication, collaboration, professionalism and leadership, are underappreciated by resident and supervisor [5–7]. Moreover, the busy workplace with its high clinical burden leaves little time for reflection on performance and for defining developmental learning objectives and activities [8–10].

Due to the lack of focus on these generic compentencies during residency, newly trained consultants often feel insufficiently prepared for practice [11, 12]. Additionally, unpreparedness was discovered to be related to burnout [12], making the importance of addressing generic competencies during residency indispensable. Embedding the development of generic competencies and reflective and deliberate practice, will better prepare the future workforce for their task.

When it comes to fostering reflection in medical students, Driessen et al. [13] provided recommendations based on the reflection-model of Korthagen. They outlined key elements for the reflection cycle: gathering information on performance, self-assessment, analysis and the creation of learning objectives [13]. Several learning methods to engage residents in reflective practice have been reported in literature: e.g. mentoring- and discussion groups, one-on-one dialogues or writing exercises.

Another promising method in postgraduate medical education could be a development-oriented performance assessment (PA), in psychology-literature referred to as an Assessment Center or Development Center. An Assessment Center is a highly structured method for performance assessment [14, 15]. It involves a test-day where the results from a number of assessment instruments, e.g. personality questionnaire, interview, group discussion, work simulation, are combined to provide insight into the actual competence levels of the participant [14, 15]. The assessor is usually a psychologist. The method has a high criterion-related predictive validity for work performance [14] and is widely used in the decision-making process during the recruitment period. As the method provides information on strengths and weaknesses, which is developmentally relevant, the method can be applied for developmental purposes as well [14, 15].

PA’s have a formative goal. They encourage reflection on performance outside the busy workplace [14, 15] and have a long range perspective with a specific focus on personal development and career planning. As such, they aim to establish new development tracks connected to the work setting, i.e. deliberately planned learning activities [14, 15]. With the use of PA’s, an independent facilitator (psychologist) provides participants with specific and detailed feedback on their performance [14]. The method is best integrated with a series of other workplace performance assessment instruments, thus establishing an integrated approach to assessment [15]. Additionally, organisational support to carry out developmental plans is essential for the effectiveness of the PA [14].

In this study we explored the potential added value of a PA into postgraduate training. We aimed to gain insight into the experiences of residents with a PA and to evaluate the effects of this intervention by conducting an exploratory qualitative study using semi-structured interviews.

## Methods

### Setting

We conducted our study amongst residents trained in one university hospital and two associated teaching hospitals in the Netherlands. Postgraduate training programmes in the Netherlands last four to six years and consist of rotations in both university- and associated teaching hospitals. All Dutch postgraduate training programmes are competency-based using the CanMEDS framework [16]. Performance progress is recorded in an electronic portfolio, based on assessments instruments such as: objective structured clinical examinations, direct observations of procedures, multisource feedback and individual learning plans. Regular progress meetings between programme director and resident take place, from four times a year in the first year to once a year during the last years of residency.

Increasingly, training programmes offer residents the opportunity to design postgraduate training according to their individual ambitions and achievements, either in a subspecialty or in specific professional ‘themes’ such as e.g. Medical Education, Management & Leadership or Patient Safety. This curricular customization is usually integrated in the last two years of residency.

### Participants

Participants in our study were residents in Obstetrics and Gynaecology, Internal Medicine, Orthopaedic Surgery and Radiology. We purposefully sampled these specialties to ensure inclusion from both medical- and surgical specialties. Residents were in their third, fourth or fifth postgraduate year (PGY) and on the verge of making profiling-choices.

Between December 2012 and April 2014 we invited twenty residents to participate. They were contacted through email sent by a third party, i.e. the administrative assistants of the departments involved. After showing initial interest, they received additional written information on the study and the PA. Participation was voluntary and withdrawal was possible at any time. We obtained informed consent from nineteen residents of whom eighteen conducted the PA. Of these eighteen, two residents did not respond to the interview invitation for reason unknown. Sixteen residents were available for the interview.

The Ethical Review Board of the Dutch Association of Medical Education approved the study (number ERB186). As this study did not receive any funding, the facilitator was paid to conduct the PA (€1000 per resident) from the regular budget for postgraduate medical training.

### The process of the development-oriented performance assessment

**Fig. 1:**
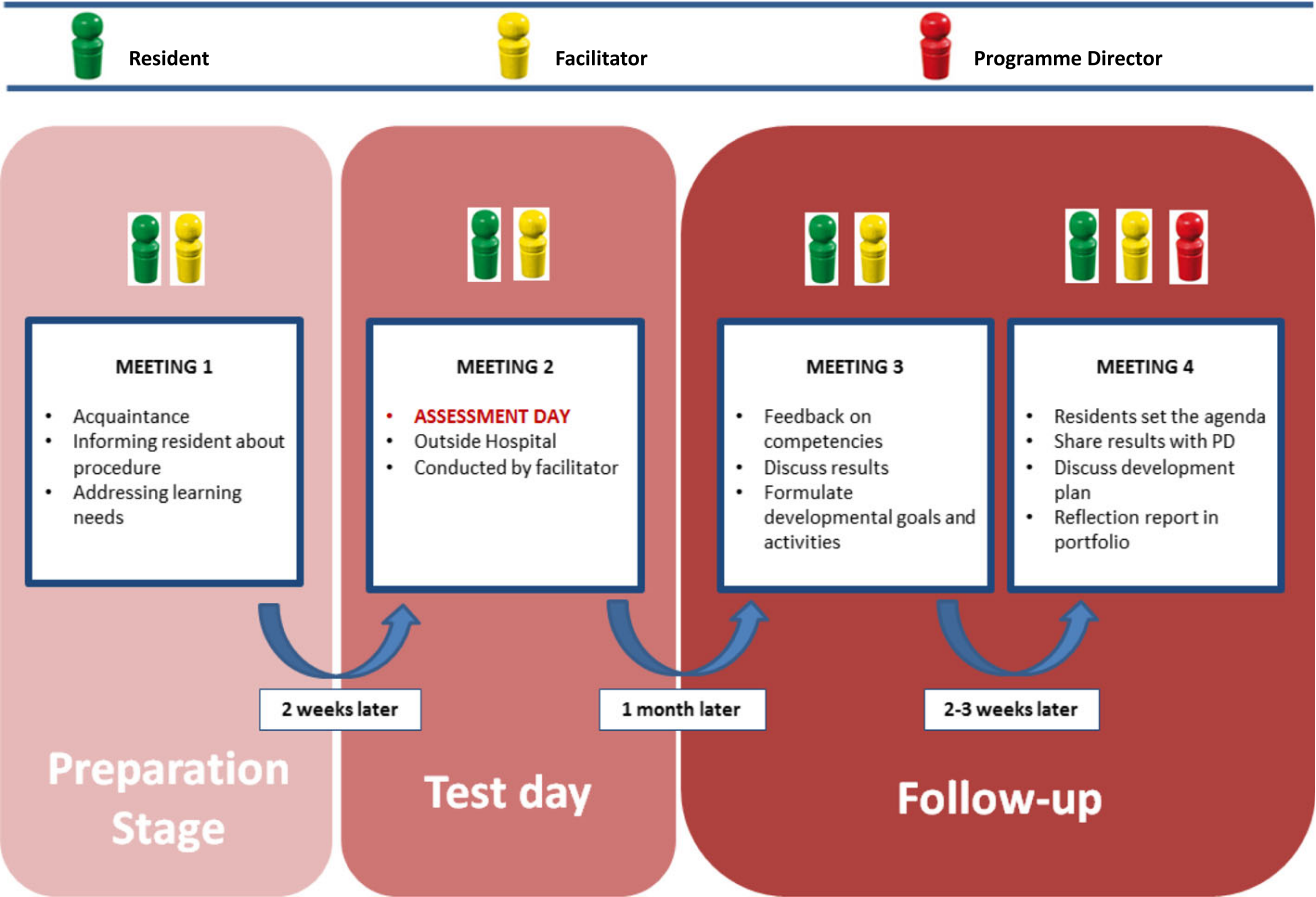
Process of the Development-oriented Performance Assessment

The basic principles for the PA were: 1) formative assessment of the individual resident conducted by an independent facilitator (i.e. psychologist-assessor), 2) focus on personal development of generic competencies, 3) participation without summative consequences for specialty training, 4) safe environment away from the workplace and 5) confidentiality between assessor, program director and resident. The following paragraphs describe the different meetings of the PA (Fig. 1).

#### Meeting 1. Getting acquainted (resident and facilitator)

Resident and facilitator met at the resident's working location. The facilitator informed the resident about the procedure of the PA. They discussed the resident’s learning objectives and learning needs in order to ascertain that these would be addressed during the assessment-day. The facilitator emphasized the formative aspect, his independency and the confidential nature of the PA. Due to the confidential nature of the PA, program directors were not allowed access to the findings.

#### Meeting 2. The performance assessment-day (resident and facilitator)

Approximately 2 weeks after the initial meeting, the facilitator conducted the performance assessment outside the hospital. The PA comprised a set of tests and interviews and was tailored to the resident’s learning needs. It focused on four CanMEDS-competencies; i.e. ‘communication’, ‘collaboration’, ‘leadership’ and ‘professionalism’. It consisted of an IQ-test, career-orientation inventory, personality inventories, self-description portrait (to assess self-reflective capacity and self-assessment) and two behavioural interviews with the facilitator. The interviews both lasted 1 hour and addressed biography, work-life balance, career-choices and career-planning, and the four competencies.

#### Meeting 3. Feedback and reflection (resident and facilitator)

The objective of this meeting was to discuss the results of the PA in a safe environment. This meeting took place at the facilitator’s premises, 1 month after the test-day. The facilitator and the resident discussed the following results: 1) intelligence, 2) personality traits, 3) an overview of strengths and weaknesses per CanMEDS-competency, 4) findings from the career-orientation inventory: description of most-valued aspects of work, e.g.: expertise, values, autonomy, wealth, creativity and 5) summary and advice. The summary contained five strengths and five developmental issues with suggestions of how to put these into practice.

After disclosing the results the facilitator discussed the feedback and ‘diagnosis for development’ with the resident. The facilitator then elaborated on the findings and clarified these where necessary. Finally, the facilitator supported the resident in reflecting on the feedback and formulating a development plan.

#### Meeting 4. Sharing results with Programme director and formulating developmental activities. (resident, facilitator and Programme director, 1 h)

The objective of this meeting was to: 1) create a safe environment in which residents shared findings of the PA with their programme director and 2) to achieve an alliance between resident and programme director regarding the development plan. The meeting took place at the resident’s working location, 2 to 3 weeks after meeting three. This time interval was chosen to create opportunity for the resident to reflect on the findings and to set the agenda for the conversation with the programme director. At this meeting the resident and program director discussed the developmental needs and the developmental plan with learning activities. The learning activities were formulated according to the SMART-framework, i.e. specific, measurable, attainable, relevant and time-framed. It was up to the resident to decide which PA-results to share with their programme director. The resident and programme director together explored possibilities to put plans into practice. The role of the programme director was to facilitate the implementation of developmental plans in the workplace.

The facilitator’s presence was presumed valuable because of his independent role and mediating potential in case of disagreement. Both facilitator and programme director reflected on the resident’s plan from their perspective.

After this last meeting, the resident added a reflection report and a personal development plan to their portfolio.

### Data collection: Interviews

Six months after meeting four, one of the researchers (SV) conducted semi-structured individual interviews with the residents. These interviews focused on preparation and expectation of the PA, acceptability of the procedure, perceptions of the received feedback and effects of the PA. Interviews lasted 45 min on average, were audio-recorded and transcribed verbatim.

### Data analysis

Two researchers analysed the transcripts (KD & JB) using the framework method for qualitative analysis [17]. The following steps were taken: 1) transcription, 2) familiarization with the interviews, 3) inductive coding: the researchers started with initial open coding the first five interviews; codes were not pre-defined by an existing theory, 4) development of a working analytical framework: codes were grouped together into categories after consensus between the two researchers was reached, 5) application of the framework: subsequent interviews were coded by applying the framework and using existing categories and codes, 6) charting of the data into the framework matrix; this involved summarizing the data from each interview into the framework matrix with references to quotations, 7) interpretation of the data; this iterative process was performed by three researchers (KD, JB & AJdB) and went through several cycles in which higher order themes and relationships between categories were identified from the data. In a final step the results were discussed with all authors.

## Results

Three major themes emerged from our data; Time for and timing of reflection, Awareness and self-confidence and Trust and commitment. The latter contained two sub-themes: Role of the facilitator and Resident and programme directors relationship.

Table 1 shows the characteristics of participating residents.

**Table 1:** Characteristics of participating residents

Characteristics	Results
Median age (range), y	31 (28–34)
Sex, n (%)	
Female	9 (56%)
Male	7 (44%)
Specialty, n (%)^a^	
Internal Medicine	4 (25%)
Obstetrics and Gynaecology	5 (31%)
Orthopaedic Surgery	3 (19%)
Radiology	4 (25%)
Postgraduate Year (PGY), n (%)	
PGY 3	7 (43,75%)
PGY 4	7 (43,75%)
PGY 5	2 (12,5%)

^a^Specialty (length of training program): Internal Medicine (6 years), Obstetrics and Gynaecology (6 years), Orthopaedic Surgery (6 years), Radiology (5 years)

### Time for and timing of reflection

Participating in the PA stimulated residents to reflect, even *before* the performance-assessment day. They mentioned having a moment of reconsideration prior to the PA, in which they reflected on their competencies, career ambitions and opportunities.

Most residents appreciated this effect of the PA, as busy clinical practice leaves little time for reflection. Participating in the PA offered a legitimate and dedicated time-out and an opportunity for contemplation.

*“It’s very easy to go with the flow during your training period; just working your way through and completing the necessary… But the PA made me think about my competencies and how to shape my personal training schedule.”* [Resident 7].

Concerning the question of when to implement the PA, residents mentioned several aspects to optimize timing. Firstly, they felt they needed experiences to reflect on. Having faced some difficult- or challenging situations during work was perceived useful in order to gain self-knowledge and overcome concerns about just medical content. Residents stated that they would not be ready for this kind of reflection in an earlier stage of residency education.

*“you are still too impressed by all the specific medical aspects of residency and you lack an overview of your own career and future”.* [Resident 2].

Secondly, a period of transition within the residency rotations was seen as effective for the PA. In our study this referred to taking imminent career decisions. Thirdly, they should have enough remaining years of postgraduate training to realize their developmental goals.

*“Timing was perfect to me; because I was in a phase in which I’d seen most of the general stuff. Now I was up for contemplating my future and the steps needed to achieve my goals….. I was able to turn my intentions into practice. This led to an actual change of my training schedule.”* [Resident 10].

### Awareness and self-confidence

By participating in the PA residents increased awareness of their competencies and learning needs. It appeared that several mechanisms contributed to this awareness. For some residents, the facilitator “*holding up a mirror to them”* [Resident 15] helped to clarify. Others described it as *“a confrontation”*[Resident 3] in which they had to be open to very personal feedback*.* Although they were not familiar with this approach, they were aware of the relevance.

One resident mentioned that the facilitator instantly identified his ‘weakness’. Through the manner of the facilitators questioning he realised the potential impact of this weakness on his performance. He did not experience this as blaming and shaming, but rather as an opportunity for personal development.

*“He* [*the facilitator*] *instantly identified my weakpoints. He asked me things such as; ‘What is the reason for this? Do you know how this comes across to others?’ etc. Those were real eye-openers for me. But, because these seemed like workable goals, the whole PA trajectory was very appealing to me.”* [Resident 9].

Various residents mentioned a stronger sense of self-confidence after the PA. It revealed personal aspects and qualities they did not realise before. By making these qualities explicit, the residents received the reinforcement they needed.

*“The PA revealed personal qualities I might knew unconsciously already but never truly realised. It helped me to position myself and my professional identity. I really needed that support at the time.* “[Resident 10].

The PA supported residents in taking responsibility and ownership of their training and strengthened them in setting and pursuing goals. Some felt confident enough to try a new approach. Goals varied from participating in specific courses; e.g. (time)-management, leadership, to changing their training schedule, switching hospital or taking part in a coaching track.

*“I wouldn’t have started the coaching track if it wasn’t for the PA. That was part of the outcome. I probably would have lingered on. But I believe I’m a better doctor now.”* [Resident 9].

### Trust and commitment

Residents acknowledged their willingness to reveal vulnerability towards both facilitator and programme director as a pivotal feature for success of the intervention. Some addressed sensitive issues and concerns during the PA. Sharing these was perceived to influence outcome of the PA in a positive way. Although the PA was work-related, residents experienced it as a deeply personal matter where openness was seen as essential.

#### Role of the facilitator

A number of characteristics were regarded as important for the credibility and appreciation of the facilitator and the way his or her feedback was accepted by residents. Residents appreciated the facilitator’s objectivity, independence and the assessment without any prior knowledge of their successes or failures. Confidentiality was considered particularly important, as this made the residents free to discuss any topic without worrying about potential consequences. The external location of the PA contributed to this feeling.

*“I appreciated the external location of the PA; it made me feel out of my role, away from the buzzer, away from my doctors coat.”* [Resident 10].

Residents experienced the fact that the facilitator was *not* a physician as beneficial since the facilitator looked through a different lens. The specific expertise regarding assessment of generic competencies was respected, in particular because the residents considered supervisors less equipped to assess these competencies.

*“It was a pleasure to have a more competency-based conversation. In regular progress-conversations we’d quickly change the focus to technical skills. But now we focused on interpersonal skills and my specific profile; which indicated my managerial potential”.* [Resident 7].

While it was considered as beneficial that the facilitator had an outsider’s perspective, residents felt it important for the facilitator to show familiarity with the medical domain and to understand their world.

*“He is an ‘outsider’. This I highly appreciated. Yet he’s knowledgeable enough about the medical world, the hierarchical system, to understand our [the residents] position. It is quite an extraordinary world after all”.* [Resident 4].

#### Resident and programme director’s relationship

Some important features concerning the relationship between programme director and resident emerged from the interviews. Although the PA consisted of meetings between facilitator and resident, residents mentioned the relationship of trust with their programme director as a distinct and important influence on the PA. A relationship based on trust made them more open to sharing outcomes of the PA with their programme director.

*“I think he’s a great programme director. You can discuss anything with him. And it feels safe to do so. If the relationship was suboptimal, participating in this experience might have been very different.”* [Resident 1].

While most residents considered themselves supported by their programme director in realizing their developmental goals, one example illustrates how a lack of support by the programme director can turn the PA’s positive effect into a negative experience. In this case, the resident had time-management difficulties and resident and facilitator agreed on the usefulness of a time-management course. However, the programme director disapproved of the resident’s learning objectives, leaving the resident disappointed.

“*He disagrees with me on more than one subject… But this event left a bitter aftertaste.”* [Resident 16].

It seemed that lack of support from a programme director with the PA and its possible outcomes might impede the effectiveness of the PA.

## Discussion

In this study we aimed to gain insight into the experiences of residents with a PA and to evaluate the effects of this intervention. Residents perceived the PA as a valuable addition to existing assessment-instruments. Development-oriented performance assessment serves as a tool to encourage reflective and deliberate practice in residents by fulfilling the following important conditions. It encourages and facilitates residents’ reflection and self-assessment, helps residents define deliberately planned learning activities, provides residents with meaningful informative feedback, and above all, it provides them with dedicated time to do all the aforementioned. In the postgraduate medical education workplace where learning activities are seldom deliberately planned this intervention could contribute to deliberate practice. We identified several factors modulating these effects: careful timing of the intervention, a trustworthy and capable facilitator and a committed programme director.

Our participants identified a safe learning environment as an important factor that encouraged reflection. As described in literature, trust plays a critical role in feedback interactions and the acceptance of feedback [18, 19] and its absence can pose a barrier to reflective practice [13]. Overall, residents experienced the procedure of the PA as ‘safe’. Over the course of the PA they built a relationship of trust with the facilitator which supported the discussion of sensitive matters and the acceptance of feedback. Our study shows that a relatively short period of time was sufficient for residents to build rapport with the facilitator.

Participating in the PA led to a conversation between resident and programme director about the development of generic competences. As previously reported [20, 21] residents in our study also mentioned that programme directors rarely discussed or provided feedback on generic competencies. A PA can help to initiate such conversations.

Parallel to trust and credibility, the ‘educational alliance’ between programme director and resident comes into play. Our results lend support to the framework described by Telio [18]. Telio uses the ‘therapeutic alliance’ analogy, as derived from psychotherapy literature. This alliance states that the better the supervisory relationship between learner and supervisor in medical education, the better the feedback is incorporated by the learner. Although residents only met their programme directors at the very end of the PA experience, the perceived relationship with their programme director seems to influence the reach of the PA. It influences how much residents reveal and expose their weaknesses, not only towards the facilitator but also towards their programme director in the final meeting. One specific aspect of this alliance, the programme director’s commitment to the learning and development of the resident, was mentioned by residents. Realization of residents’ ambitions and development plans depends largely on the support and commitment of the programme director. This finding is in line with previous publications on effects of a PA [14, 15], which state that without support from the organisation any positive effect that a PA might have will not be sustained.

Our study has several limitations. There is potential selection bias as all residents were self-selected volunteers. It could be that residents who would benefit the most from reflection did not participate in our study and that participating residents were the more reflective ones. Furthermore, we acknowledge that our study was conducted in just one teaching region and that reported effects are perceptions of the residents, rather than measurable outcomes. It would be relevant to research whether the PA contributes to enhanced self-assessment and sustained reflective and deliberate practice in a larger setting, spanning multiple regions. Future studies might look into these effects more deeply.

An important aspect to take in mind, when considering implementation of a PA, are the expenses. We hired an external professional facilitator to conduct the PA and payed the accompanying fee from postgraduate training budget. Whether the benefits of structural usage outweigh the expenses of this method could be investigated in a return on investment study.

We found the application of the PA during the residency period feasible and we expect this experience to be similar during other period over the medical education continuum. In our study we tailored the PA to focus on generic competencies, reflective and deliberate practice and career-planning in residents who were halfway their postgraduate training. However, a PA might serve different purposes at different times. For example, it might be valuable in later career stages as a part of continuous professional development.

## Conclusion

Collectively, from our findings we can state that the value of a PA halfway through the medical residency lies in its ability to engage residents in meaningful reflection and deliberate practice, in its enhancement of the professional development of generic competencies and in its shift of focus towards a resident’s future career. According to residents, the PA’s approach is truly different from other workplace-based assessments and adds value to the existing assessment-instruments. Benefits for the residents are an increased awareness of competencies, self-confidence and feelings of career-ownership. Key-elements for successful implementation are a safe learning- and assessment-environment outside the clinical workplace, an expert facilitator and support from a committed programme director to pursue developmental goals.

## Abbreviations

PA: Development-oriented performance assessment
PD: Programme director
